# Facial pain, health-related quality of life and trismus-related symptoms up to 5 years post-radiotherapy for head and neck cancer

**DOI:** 10.1007/s00520-023-08162-y

**Published:** 2023-11-15

**Authors:** Susan Aghajanzadeh, Therese Karlsson, Lisa Tuomi, My Engström, Caterina Finizia

**Affiliations:** 1 Department of Otorhinolaryngology, Head and Neck Surgery, Institute of Clinical Sciences, Sahlgrenska Academy, University of Gothenburg, 413 45 Gothenburg, Sweden; 2 Region Västra Götaland, Department of Otorhinolaryngology- Head & Neck Surgery, Sahlgrenska University Hospital, Gothenburg, Sweden; 3 Institute of Neuroscience and Physiology, Speech and Language Pathology Unit, Sahlgrenska Academy, University of Gothenburg, Gothenburg, Sweden; 4 Institute of Health and Care Sciences, Sahlgrenska Academy, University of Gothenburg, Gothenburg, Sweden; 5 Department of Surgery Gothenburg, Region Västra Götaland, Sahlgrenska University Hospital, Gothenburg, Sweden

**Keywords:** Facial pain, Head and neck cancer, Trismus, Radiation therapy, Health-related quality of life

## Abstract

**Purpose:**

Pain is a frequent symptom of head and neck cancer (HNC) but longitudinal studies investigating facial pain are scarce. We aimed to investigate prevalence of facial pain, its effect on health-related quality of life (HRQL) and trismus-related symptoms in a HNC cohort.

**Methods:**

Patients (*n* = 194) were prospectively followed post completion of radiotherapy (RT). Outcome measures included facial pain, HRQL, trismus-specific symptoms, and maximal interincisal opening (MIO).

**Results:**

Facial pain was reported by 50% at baseline. Corresponding figures for 3-, 12-, and 60 months post-RT were 70%, 54% and 41%. Moderate to severe pain was reported in 29–44% of patients reporting pain during the study period. Patients reporting pain scored significantly worse on more HRQL variables and trismus symptoms, as well as had significantly smaller MIO at all follow-up time points.

**Conclusions:**

Facial pain was common in HNC patients pre- and post-RT and remained prevalent up to 5 years after completion of RT. Reductions in MIO were associated with more facial pain. Pain was also associated with worse HRQL.

## Introduction

Head and neck cancer (HNC) is the fifth fastest increasing cancer type in Sweden 1 and accounts for over 900,000 new cases annually worldwide 2. Approximately half of the patients with HNC present with late tumor stages, i.e., T3 or T4 3. Treatment strategies for HNC include surgery, radiotherapy (RT) and chemotherapy. In early-stage HNC, unimodal therapy is common, whilst multimodal approaches are usually indicated in more advanced stages 4.

Pain is a common symptom among HNC patients, either due to the cancer itself or as a result of acute or late adverse effects of treatment [5–7. Cancer-related pain after treatment has been associated with negative effects on quality of life 8. Furthermore, trismus is another burdensome complication that may occur in up to 40% of patients with HNC after oncological treatment [9–11. Trismus, defined as a maximal interincisal opening (MIO) of ≤ 35 mm 12, has been associated with pain, decreased health-related quality of life (HRQL), as well as eating difficulties and maintaining oral hygiene 13, 14. Radiation fibrosis of the masticatory muscles is thought to be the primary cause of postradiation trismus 15, 16.

Few studies have to date described radiation-induced trismus and its association to pain longitudinally. The longest thus far is by this research group, for up to 3 years after completion of radiotherapy in a cohort half the size of the current study 17, 18. The study found facial pain to be reported by over half of the HNC cohort before oncological treatment and at 3 years post radiotherapy. Patients reporting facial pain at the 3-year follow-up had a lower MIO than the patients that did not suffer from facial pain. Other studies have reported on oral pain specifically, with cross-sectional or retrospective study designs 19, 20 and hence, studies investigating long-term prevalence of pain in patients with HNC after treatment are scarce [21–23.

As the prognosis of HNC is improving with modern oncological treatments and a large subgroup of HNC are expected to live many years after their cancer treatment, research focusing on mapping pain and improving HRQL after HNC treatment is warranted. Hence, the aim of this prospective observational study is to investigate the prevalence of facial pain, as well as its effect on HRQL and trismus-specific symptoms in patients with HNC for 5 years post-RT. A secondary aim includes investigating the correlation between facial pain and the degree of maximal interincisal opening.

## Materials and methods

### Study participants and design

Participants were recruited from the weekly multidisciplinary tumor board meeting at Sahlgrenska University Hospital in Gothenburg, Sweden between 2007 and 2012. Patients with newly diagnosed HNC that fulfilled the inclusion criteria were consecutively invited for study participation. Inclusion criteria included age ≥ 18 years, planned for primary radiotherapy with curative intent and tumor site in oral cavity, oropharynx, nasopharynx or cervical carcinoma of unknown primary (CCUP). Since the cohort was going to be used for multiple studies regarding postradiation trismus, additional exclusion criteria were applied, i.e., the presence of trismus prior to treatment and a tumor site where one would not normally expect trismus as a complication (larynx, esophagus, hypopharynx and skin malignancy). Patients were also excluded if they had recurrent disease, surgical treatment only, insufficient Swedish language skills and performance status or mental capacity too poor to partake in examinations or respond to questionnaires. Patients were followed prior to radiotherapy (baseline), 3 months, 12 months, and 60 months post-radiotherapy completion.

### Oncologic treatment

Traditional external radiotherapy with curative intent was given according to regional guidelines. Between the years 2007 and 2009, accelerated hyperfractionated radiotherapy was administered to patients (about half of the patients), consisting of 1.7 Gray (Gy) doses given twice daily, 5 days/week (total radiation dose of 64.6 Gy). During the time period 2010–2012 however, the other half of patients received accelerated fractioning 1–2 times daily in 2–2.4 Gy fractions, 5 days/week (total dose was 64–68 Gy). Sixty-two percent (*n* = 121), mainly those with advanced tumor stages, also received induction (cisplatin and 5-fluorouracil) or concomitant (weekly cisplatin) chemotherapy according to regional guidelines at the time. For oropharyngeal and nasopharyngeal cancers, non-surgical oncological treatment regimens were used.

Advanced stage (III–IV) oral tumors were typically also treated with surgical excision, including neck dissections. In cases where the tumor was assessed to be unresectable, chemoradiotherapy was administered instead. Other tumor sites where surgical removal was performed including neck dissections were salivary gland cancers and sinonasal cancers. In selected CCUP cases, neck dissection was performed prior to non-surgical oncological treatment.

### Data collection and measurement tools

#### Adult Comorbidity Evaluation 27 (ACE-27)

Adult Comorbidity Evaluation 27 (ACE-27) was recorded at baseline. The ACE-27 is a validated comorbidity instrument widely used in head and neck oncology. It includes the assessment of 27 elements from twelve organ systems. Based on the amount and severity of comorbidities, it provides an overall comorbidity score of 0–3, where 3 indicates maximum severity 24.

#### Maximum interincisal opening (MIO)

MIO was measured in millimeters with a ruler and registered at each time point. A maximal interincisal distance of ≤ 35 mm as proposed by Dijkstra et al. 12 was used for definition of trismus.

#### The European Organization for Research and Treatment of Cancer (EORTC) Quality-of-Life Questionnaire Core-30 (QLQ-C30) and Head and Neck 35 (HN35)

The EORTC QLQ-C30 was developed for assessing the quality of life of cancer patients. It is comprised of multi-item scales as well as single items, including five functional scales, three symptom scales, a scale regarding global health and quality of life and six single items 25.

An additional Head and Neck disease specific module, the EORTC QLQ-HN35 was also utilized. The EORTC QLQ-HN35 consists of seven multi-item scales assessing pain, swallowing, senses, speech, social eating, social contact and sexuality in addition to 11 single items 26, 27.

For the EORTC questionnaires, a 4-point Likert scale was used, and scores are then transformed to a scale from 0 to 100, where high points correspond to high function on the functioning scales and Global HRQL, whilst high points correspond to a higher symptom burden on the symptom scales and items 26, 27.

#### Gothenburg Trismus Questionnaire (GTQ)

The GTQ is a validated trismus-specific questionnaire, developed to be used as a complement to the objective measure MIO and was registered at all study time points. It is composed of 21 items in total, where 13 items are incorporated into the domains jaw-related problems (7 items), eating limitations (4 items), muscular tension (2 items) and the remaining single 8 items assess facial pain, pain associated with trismus, trismus affecting work, leisure or social life. In the GTQ, eight items in total are directly related to facial pain. Score ranges from 0 to 100, where a high score indicates a high symptom burden 28.

The primary variable “Intensity of facial pain during the last month” was reported on a 7-point Likert scale where 1 point equals no facial pain and 7 points equates to unbearable pain. Furthermore, 2–3 points are considered mild pain, 4 points moderate pain and 5–6 points severe pain. The GTQ has been previously described in detail 29. Patients were subgrouped into two groups: those reporting “No pain” (1 point) and those reporting some degree of “Pain” (2–7 points) on the GTQ variable “Intensity of facial pain during the last month”.

### Statistical analyses

The GTQ variable “Intensity of facial pain during the last month” was dichotomized with the purpose of distinguishing those that had some degree of facial pain from completely pain-free patients. Standard procedures were used to calculate descriptive statistics with 95% confidence interval. The SAS version 9.4 for PC was used for analyses. For comparisons between groups, Fisher’s Exact test was used for dichotomous variables, Mann–Whitney *U*-test for continuous variables, Mantel–Haenszel Chi Square test for ordered categorical variables, and Chi Square test for non-ordered categorical variables. Wilcoxon signed rank test was used for continuous variables within groups over time. Spearman’s correlation test was used to analyze the correlation between the degree of pain and MIO. Cohens’ convention was used for interpretation of correlations. A correlation coefficient of 0.10 represents a weak or small association, 0.30 is considered a moderate correlation and 0.50 or larger represents a strong or large correlation 30. All tests are two-tailed and conducted at a 5% significance level.

### Ethical considerations

This prospective study was conducted in accordance with the Declaration of Helsinki and was approved by the Regional Ethic Review Board in Gothenburg, Sweden. All participants gave their written informed consent before study inclusion.

## Results

### Patient demographics and treatment characteristics

Demographic data and treatment characteristics of the patient cohort stratified by reporting “No pain” versus “Pain” during the last month at each time point are described in Table 1. At baseline, the median age for the whole cohort (*n* = 194) was 61 years. The subjects were predominantly male (74%), currently non-smokers (77%), and the most common primary site of tumor was oropharyngeal (63%). In total, 70% of subjects presented with advanced stage disease and 61% received combination treatment with RT and chemotherapy. When comparing the groups at baseline, twice as many women were seen in the “Pain”-group than in the “No pain”-group (32% versus 16%, respectively). Furthermore, at baseline there were more smokers in the” Pain”-group (32% versus 14%). This difference was statistically significant (*p* =  < 0.01) and similar results regarding smoking was seen at the 12-month follow-up time point (*p* =  < 0.001). Regarding differences in BMI, a statistically significant difference (*p* =  < 0.01) was seen between the “No Pain” and “Pain” groups only at the 5-year follow-up where the “Pain”-group had higher BMI scores.

**Table 1:** Sociodemographic data at baseline (pre-radiotherapy), 3 months post-radiotherapy (post-RT), 12 months post-RT, and 60 months post-RT by Pain vs no Pain

	Baseline (*n* = 194)			3 months post-RT (*n* = 185)			12 months post-RT (*n* = 168)			60 months post-RT (*n* = 126)		
	No pain (*n* = 97)	Pain (n = 97)	*P* value	No pain (*n* = 56)	Pain (*n* = 129)	*P* value	No pain (*n* = 78)	Pain (*n* = 90)	*P* value	No pain (n = 74)	Pain (*n* = 52)	*P* value
Age, Mean (95% CI)	61.2 (59–63)	60.6 (58–63)	0.57	62.2 (60–65)	60.7 (59–63)	0.32	60.6 (58–63)	60.1 (58–62)	0.70	60.3 (58–63)	58.3 (56–61)	0.15
Sex, n (%)			0.01			0.60			1.00			1.00
Male	81 (84)	65 (67)		38 (68)	94 (73)		59 (76)	67 (74)		52 (70)	37 (71)	
Female	16 (16)	32 (33)		18 (32)	35 (27)		19 (24)	23 (26)		22 (30)	15 (29)	
Living alone, n (%)			0.14			0.83			0.67			0.08
Yes	20 (21)	30 (31)		14 (25)	36 (28)		16 (21)	22 (24)		13 (18)	17 (33)	
No	77 (79)	67 (69)		42 (75)	93 (72)		62 (79)	68 (76)		61 (82)	35 (67)	
Educational years, n (%)			0.17			0.97			0.39			0.12
6–9 years	21 (22)	28 (29)		14 (25)	31 (24)		22 (28)	21 (23)		22 (30)	7 (13)	
9–12 years	46 (47)	46 (47)		26 (46)	63 (49)		38 (49)	44 (49)		31 (42)	28 (54)	
> 12 years	30 (31)	23 (24)		16 (29)	35 (27)		18 (23)	25 (28)		21 (28)	17 (33)	
Employment status, n (%)			0.47			0.28			0.19			0.37
Full time	45 (47)	46 (48)		21 (37)	64 (50)		37 (47)	47 (52)		35 (47)	32 (62)	
Part time	7 (7)	8 (8)		6 (11)	8 (6)		6 (8)	8 (9)		7 (10)	4 (8)	
Unemployed	4 (4)	2 (2)		3 (5)	3 (2)		1 (1)	4 (4)		3 (4)	0 (0)	
Old age pensioner	34 (35)	31 (32)		20 (36)	43 (33)		23 (30)	26 (29)		21 (28)	10 (19)	
Early retiree	7 (7)	10 (10)		6 (11)	11 (9)		11 (14)	5 (6)		8 (11)	6 (12)	
Smoking, n (%)			< 0.01			0.54			< 0.001			1.00
No	83 (86)	66 (68)		46 (82)	99 (77)		70 (90)	58 (64)		57 (77)	40 (77)	
Yes	14 (14)	31 (32)		10 (18)	30 (23)		8 (10)	32 (36)		17 (23)	12 (23)	
Karnofsky Performance Scale*, Mean (95% CI)	97.7 (97–99)	97.1 (96–99)	0.81	98.9 (98–100)	97.2 (96–98)	0.07	98.6 (98–100)	96.8 (95–98)	0.05	99.2 (98–100)	97.9 (96–100)	0.21
BMI, n (%)			0.64			0.42			0.85			< 0.01
< 18.5	1 (1)	2 (2)		1 (1)	2 (2)		1 (1)	0 (0)		2 (2)	0 (0)	
18.5–25	30 (31)	31 (32)		21 (38)	40 (31)		26 (33)	31 (34)		28 (38)	8 (15)	
> 25 Missing = 1	66 (68)	63 (66)		34 (61)	86 (67)		51 (66)	59 (66)		44 (60)	44 (85)	
TNM (UICC) stage n (%)			0.94			0.24			0.62			1.00
I	4 (4)	4 (4)		3 (5)	3 (2)		4 (5)	3 (3)		3 (4)	3 (6)	
II	13 (13)	17 (17)		11 (20)	23 (18)		12 (16)	15 (17)		16 (22)	7 (13)	
III	28 (29)	19 (20)		14 (25)	27 (21)		15 (19)	26 (29)		13 (17)	15 (29)	
IV	52 (54)	57 (59)		28 (50)	76 (59)		47 (60)	46 (51)		42 (57)	27 (52)	
Tumor site n (%)			0.02			0.90			0.71			0.70
Oropharynx	64 (66)	56 (58)		36 (64)	82 (64)		55 (70)	60 (67)		53 (72)	29 (56)	
Oral cavity	6 (6)	26 (27)		8 (14)	19 (15)		6 (8)	14 (16)		7 (9)	8 (15)	
CCUP	13 (14)	7 (7)		5 (9)	14 (11)		8 (10)	9 (10)		6 (8)	8 (15)	
Nasopharynx	5 (5)	4 (4)		4 (7)	7 (5)		5 (6)	4 (4)		4 (5)	3 (6)	
Salivary gland	5 (5)	3 (3)		1 (2)	4 (3)		2 (3)	2 (2)		2 (3)	3 (6)	
Paranasal sinus and nasal cavity	4 (4)	1 (1)		2 (4)	3 (2)		2 (3)	1 (1)		2 (3)	1 (2)	
Cancer treatment, n (%)			0.39			0.25			0.92			0.22
RT only	17 (17)	22 (23)		14 (25)	24 (19)		14 (18)	16 (18)		14 (19)	10 (19)	
Surgery + [C]RT	18 (19)	16 (16)		11 (20)	17 (13)		11 (14)	13 (14)		10 (13)	13 (25)	
CRT	62 (64)	59 (61)		31 (55)	88 (68)		53 (68)	61 (68)		50 (68)	29 (56)	
ACE27, n (%)			0.40			0.86			0.95			0.56
No comorbidity	40 (41)	41 (43)		22 (39)	60 (47)		33 (42)	41 (46)		36 (49)	27 (52)	
Mild comorbidity	44 (46)	29 (30)		27 (48)	41 (32)		33 (42)	30 (33)		28 (38)	14 (27)	
Moderate comorbidity	8 (8)	23 (24)		6 (11)	23 (18)		9 (12)	17 (19)		10 (13)	9 (17)	
Severe comorbidity	5 (5)	3 (3)		1 (2)	4 (3)		3 (4)	2 (2)		0 (0)	2 (4)	

*ACE27*, Adult Comorbidity Evaluation 27; *BMI*, Body Mass Index; *CCUP*, Cervical Carcinoma of Unknown Primary; *CI*, confidence Interval; *CRT*, chemoradiotherapy; *RT*, Radiotherapy, Range from 0 to 100 indicates perfect health; *TNM*, Tumor, Node, Metastasis; *UICC*, Union for International Cancer Control

A tumor site of oral cavity was also more common in the “Pain”-group than in “No Pain”-group at baseline (27% versus 6%).

### Prevalence of facial pain over time

Half of the cohort (*n* = 97) reported facial pain during the last month at baseline (Table 1). This number increased to 70% at 3 months post-RT and decreased to 54% at 12 months post-RT. At 60 months post-RT, 41% of the subjects reported some degree of pain. When comparing no reported pain versus some degree of reported pain at each follow-up time point, there was a statistically significant difference between the groups, only at 3 months post-RT (*p* ≤ 0.001).

### Patient-reported outcome measures over time

Tables 2, 3, and 4 present the results from the EORTC QLQ-C30, HN35 and GTQ at all four study time points for both groups.

**Table 2:** EORTC QLQ C30 at baseline (pre-radiotherapy), 3 months post-radiotherapy (post-RT), 12 months post-RT, and 60 months post-RT for HNC patients with and without pain

	Baseline Mean (95% CI)*			3 months post-RT			12 months post-RT			60 months post-RT		
	No pain (*n* = 97)	Pain (*n* = 97)	*P* value	No pain (*n* = 56)	Pain (*n* = 129)	*P* value	No pain (*n* = 78)	Pain (*n* = 90)	*P* value	No pain (n = 74)	Pain (*n* = 52)	*P* value
Functioning domains												
Physical function	93.6 (91–97)	87.4 (84–91)	< 0.001	86.4 (82–91)	74.8 (71–79)	< 0.001	90.6 (87–94)	82.2 (79–86)	< 0.001	88.2 (84–93)	78.3 (72–85)	< 0.01
Role function	84.9 (79–91)	65.3 (58–72)	< 0.001	76.5 (69–84)	55.5 (50–62)	< 0.001	87.7 (83–93)	67.6 (61–74)	< 0.001	89.7 (85–94)	71.8 (62–81)	< 0.001
Emotional function	76.5 (72–81)	64.3 (60–69)	< 0.001	87.3 (83–92)	72.6 (69–77)	< 0.001	91.7 (89–95)	72.0 (67–77)	< 0.001	91.8 (88–95)	73.6 (67–80)	< 0.001
Cognitive function	87.5 (84–91)	82.1 (78–86)	0.01	87.9 (83–93)	79.0 (75–83)	< 0.01	91.5 (88–95)	79.0 (74–84)	< 0.001	88.7 (85–92)	76.6 (70–83)	< 0.01
Social function	86.6 (82–91)	78.8 (74–84)	< 0.01	77.6 (70–85)	64.6 (59–70)	< 0.01	88.0 (83–93)	73.1 (67–79)	< 0.001	89.9 (85–95)	75.6 (68–83)	< 0.001
Global QoL	70.8 (66–75)	62.2 (58–66)	< 0.01	67.9 (63–73)	58.2 (55–62)	< 0.01	75.2 (71–80)	62.3 (58–67)	< 0.001	78.7 (74–84)	62.0 (55–69)	< 0.001
Symptom domains												
Fatigue	15.7 (12–20)	30.1 (26–35)	< 0.001	27.2 (21–34)	44.4 (40–49)	< 0.001	19.1 (14–24)	33.6 (29–39)	< 0.001	17.2 (11–24)	34.6 (27–43)	< 0.001
Nausea /Vomiting	2.9 (1–5)	6.1 (3–9)	0.01	4.8 (2–8)	11.5 (8–15)	< 0.01	3.0 (1–5)	6.2 (3–9)	0.11	1.6 (0–3)	7.1 (2–12)	< 0.01
Pain	13.4 (8–18)	33.7 (28–39)	< 0.001	11.3 (6–16)	33.1 (29–38)	< 0.001	8.1 (4–12)	27.0 (22–32)	< 0.001	7.5 (3–12)	30.8 (22–40)	< 0.001
Single items												
Dyspnoea	9.4 (5–14)	22.1 (17*–*28)	< 0.001	13.1 (8–19)	34.6 (30–40)	< 0.001	12.8 (8–17)	22.5 (17–28)	< 0.001	11.0 (6–16)	32.1 (25–39)	< 0.001
Insomnia	24.4 (18–30)	32.6 (27–39)	0.03	13.7 (7–20)	34.4 (29–40)	< 0.001	8.6 (4–13)	29.5 (22–36)	< 0.001	14.1 (8–20)	34.6 (26–43)	< 0.001
Appetite loss	10.0 (5–15)	15.2 (10–20)	0.05	34.0 (23–44)	39.1 (33–45)	0.32	11.1 (6–16)	28.8 (22–36)	< 0.001	4.2 (0–8)	16.7 (10–23)	< 0.001
Constipation	5.8 (3–9)	9.1 (5–14)	0.17	11.9 (6–18)	20.1 (15–25)	0.06	8.1 (4–13)	12.0 (8–17)	0.14	5.6 (2–9)	17.3 (10–25)	< 0.01
Diarrhoea	2.8 (1–5)	5.9 (3–8)	0.07	6.2 (2–10)	7.9 (5–11)	0.53	2.1 (0–4)	8.3 (4–13)	0.02	4.3 (1–8)	7.7 (2–14)	0.37
Financial loss	8.1 (4–12)	19.8 (14–26)	< 0.01	14.8 (8–22)	27.4 (22–33)	0.01	9.4 (4–15)	24.6 (18–31)	< 0.001	3.3 (0–6)	18.6 (10–27)	< 0.001

*The data are displayed as mean (95% *CI*, Confidence Interval)

**Table 3:** EORTC QLQ HN35 at baseline (pre-radiotherapy), 3 months post-radiotherapy (post-RT), 12 months post-RT, and 60 months post-RT for HNC patients with and without pain

	Baseline Mean (95% CI)*			3 months post-RT			12 months post-RT			60 months post-RT		
	No pain (*n* = 97)	Pain (*n* = 97)	*P* value	No pain (*n* = 56)	Pain (*n* = 129)	*P* value	No pain (*n* = 78)	Pain (*n* = 90)	*P* value	No pain (*n* = 74)	Pain (*n* = 52)	*P* value
Symptom domains												
Pain	12.6 (9–16)	30.8 (26–35)	< 0.001	18.8 (14–24)	38.6 (34–43)	< 0.001	11.9 (8–15)	33.0 (29–38)	< 0.001	7.6 (5–10)	29.6 (23–37)	< 0.001
Swallowing	10.6 (7–15)	18.2 (14–23)	< 0.01	23.2 (17–29)	33.1 (28–38)	0.03	15.6 (11–20)	27.1 (22–32)	< 0.001	11.5 (7–16)	27.2 (19–35)	< 0.001
Senses	8.4 (5–12)	9.0 (5–13)	0.95	31.5 (25–39)	40.2 (35–46)	0.10	22.9 (18–28)	35.9 (30–42)	< 0.01	15.3 (10–21)	31.0 (23–39)	< 0.001
Speech	9.1 (6–12)	15.7 (11–20)	0.02	12.9 (9–16)	24.9 (21–28)	< 0.001	8.4 (5–11)	22.4 (18–27)	< 0.001	7.4 (4–11)	22.9 (16–30)	< 0.001
Social eating	8.4 (5–11)	17.5 (13–22)	< 0.001	29.7 (22–37)	39.0 (34–44)	0.04	18.1 (13–23)	35.8 (29–43)	< 0.001	11.3 (7–16)	26.4 (19–34)	< 0.001
Social contact	5.3 (3–8)	9.3 (6–13)	0.06	5.7 (2–9)	14.4 (11–18)	< 0.001	3.9 (1–6)	13.7 (9–18)	< 0.001	3.5 (2–5)	16.2 (10–23)	< 0.001
Sexuality	23.3 (16–30)	34.1 (26–42)	0.03	35.0 (25–45)	51.8 (45–59)	< 0.01	21.8 (15–29)	41.2 (33–49)	< 0.001	29.0 (21–37)	37.8 (28–48)	0.11
Symptoms single items												
Teeth	2.8 (1–5)	11.1 (6–16)	< 0.01	7.1 (2–12)	21.1 (16–26)	< 0.001	13.0 (7–19)	27.0 (21–34)	< 0.001	13.1 (7–19)	35.9 (25–47)	< 0.001
Opening mouth	2 (0–5)	14.2 (9–20)	< 0.001	16.1 (10–22)	28.4 (23–34)	< 0.01	11.1 (7–15)	34.1 (28–40)	< 0.001	9.3 (4–14)	39.7 (30–49)	< 0.001
Dry mouth	13.7 (9–19)	20.7 (15–26)	0.02	68.5 (60–77)	77.2 (72–82)	0.07	57.6 (50–65)	74.9 (70–81)	< 0.001	46.9 (40–54)	67.3 (58–76)	< 0.001
Sticky saliva	13.7 (9–19)	18.7 (14–24)	0.05	57.0 (47–67)	58.8 (53–65)	0.78	48.5 (41–56)	61.4 (54–69)	0.02	32.9 (25–41)	62.7 (49–77)	< 0.001
Cough	12.0 (8–16)	21.9 (17–27)	< 0.01	20.8 (14–28)	27.6 (23–33)	0.15	16.0 (10–22)	25.8 (20–31)	< 0.01	14.4 (9–20)	34.0 (25–43)	< 0.001
Felt ill	13.7 (9–19)	23.9 (18–30)	< 0.01	11.9 (7–17)	28.3 (23–34)	< 0.001	7.8 (4–12)	23.5 (17–30)	< 0.001	3.7 (1–6)	23.7 (16–32)	< 0.001
Pain killers	40.2 (30–50)	64.2 (54–74)	< 0.001	28.6 (16–41)	60.6 (52–69)	< 0.001	20.5 (11–30)	45.6 (35–56)	< 0.001	23.6 (13–34)	38.5 (25–52)	0.06
Nutritional supplement	9.3 (3–15)	15.8 (8–23)	0.17	39.3 (26–53)	48.0 (40–57)	0.28	21.8 (12–31)	24.4 (15–34)	0.69	12.5 (5–20)	23.1 (11–35)	0.12

*The data are displayed as mean (95% *CI*, Confidence Interval)

**Table 4:** GTQ at baseline (pre-radiotherapy), 3 months post-radiotherapy (post-RT), 12 months post-RT, and 60 months post-RT for HNC patients with and without pain

	Baseline Mean (95% CI)*			3 months post-RT			12 months post-RT			60 months post-RT		
	No pain (*n* = 97)	Pain (*n* = 97)	*P* value	No pain (*n* = 56)	Pain (*n* = 129)	*P* value	No pain (*n* = 78)	Pain (*n* = 90)	*P* value	No pain (*n* = 74)	Pain (*n* = 52)	*P* value
Domains												
Jaw-related problems	2.4 (1–4)	16.9 (13–21)	< 0.001	10.2 (7–13)	32.5 (29–37)	< 0.001	10.3 (7–13)	37.7 (33–42)	< 0.001	9.5 (6–13)	40.5 (34–47)	< 0.001
Eating limitations	2.9 (1–5)	18.6 (13–24)	< 0.001	17.4 (11–24)	34.3 (30–39)	< 0.001	16.9 (12–22)	34.3 (29–40)	< 0.001	17.0 (11–23)	35.5 (28–43)	< 0.001
Muscular tension	4.9 (3–7)	9.3 (7–12)	< 0.01	8.2 (5–11)	20.3 (17–23)	< 0.001	9.7 (6–13)	24.9 (21–29)	< 0.001	14.8 (10–20)	35.1 (29–41)	< 0.001
Single items												
Facial pain												
Right now	1.4 (0–3)	17.2 (13–22)	< 0.001	0.9 (0–2)	20.4 (17–24)	< 0.001	0.4 (0–1)	23.3 (19–27)	< 0.001	0.9 (0–2)	27.2 (22–33)	< 0.001
Pain when worst last month	2.1 (0–4)	42.5 (38–47)	< 0.001	1.5 (0–3)	40.6 (37–44)	< 0.001	2.4 (0–4)	34.8 (31–39)	< 0.001	0.0 (0–0)	35.9 (30–42)	< 0.001
Pain interfering with social, leisure and family activities	0.5 (0–1)	12.5 (8–17)	< 0.001	0.9 (0–2)	19.9 (16–24)	< 0.001	0.7 (0–2)	15.3 (11–20)	< 0.001	1.4 (0–3)	23.6 (15–32)	< 0.001
Pain affecting ability to work last month	0.5 (0–1)	18.8 (13–25)	< 0.001	0.5 (0–1)	22.1 (17–27)	< 0.001	0.6 (0–2)	18.0 (12–24)	< 0.001	1.7 (0–4)	18.6 (12–26)	< 0.001
Limitations in mouth opening (LOM)	1.4 (0–3)	15.1 (10–20)	< 0.001	15.1 (10–20)	33.6 (29–38)	< 0.001	12.8 (9–17)	35.1 (30–41)	< 0.001	11.1 (7–16)	38.0 (31–45)	< 0.001
LOM before cancer	2.6 (0–5)	2.9 (1–5)	0.44	3.2 (0–7)	7.2 (4–11)	0.11	0.6 (0–2)	3.1 (1–5)	< 0.05	1.4 (0–3)	13.0 (6–20)	< 0.001
LOM interfering with social, leisure and family activities	0.8 (0–2)	7.4 (3–11)	< 0.01	3.2 (1–5)	16.8 (13–21)	< 0.001	4.9 (2–8)	16.3 (11–21)	< 0.001	4.1 (1–7)	21.6 (14–29)	< 0.001
LOM affecting ability to work	0.3 (0–1)	8.6 (5–13)	< 0.001	1.4 (0–3)	16.7 (12–21)	< 0.001	2.6 (0–5)	18.5 (13–24)	< 0.001	2.0 (0–4)	19.6 (12–27)	< 0.001

*The data are displayed as mean (95% *CI*, Confidence Interval)

#### The European Organization for Research and Treatment of Cancer (EORTC) Quality-of-Life

For the EORTC QLQ-C30 questionnaire, the pain group reported significantly worse scores on all the functioning scales, *Global QoL*, the majority of single items at all time-points, as well as all symptom scales except nausea/vomiting at the 12-month follow-up (*p* = 0.11) (Table 2).

The pain group also reported significantly worse scores compared to those without pain regarding most EORTC QLQ-HN35 scales and single items at 12- and 60 months post-RT and on most scales at 3 months post-RT (Table 3).

#### Gothenburg Trismus Questionnaire

Furthermore, the pain group reported significantly worse scores on all GTQ items except for *Limited mouth opening before cancer* at baseline and 3 months post-RT, and on all items at 12 months and 60 months post-RT completion (Table 4).

### MIO group comparison and pain correlation

**Fig. 1:**
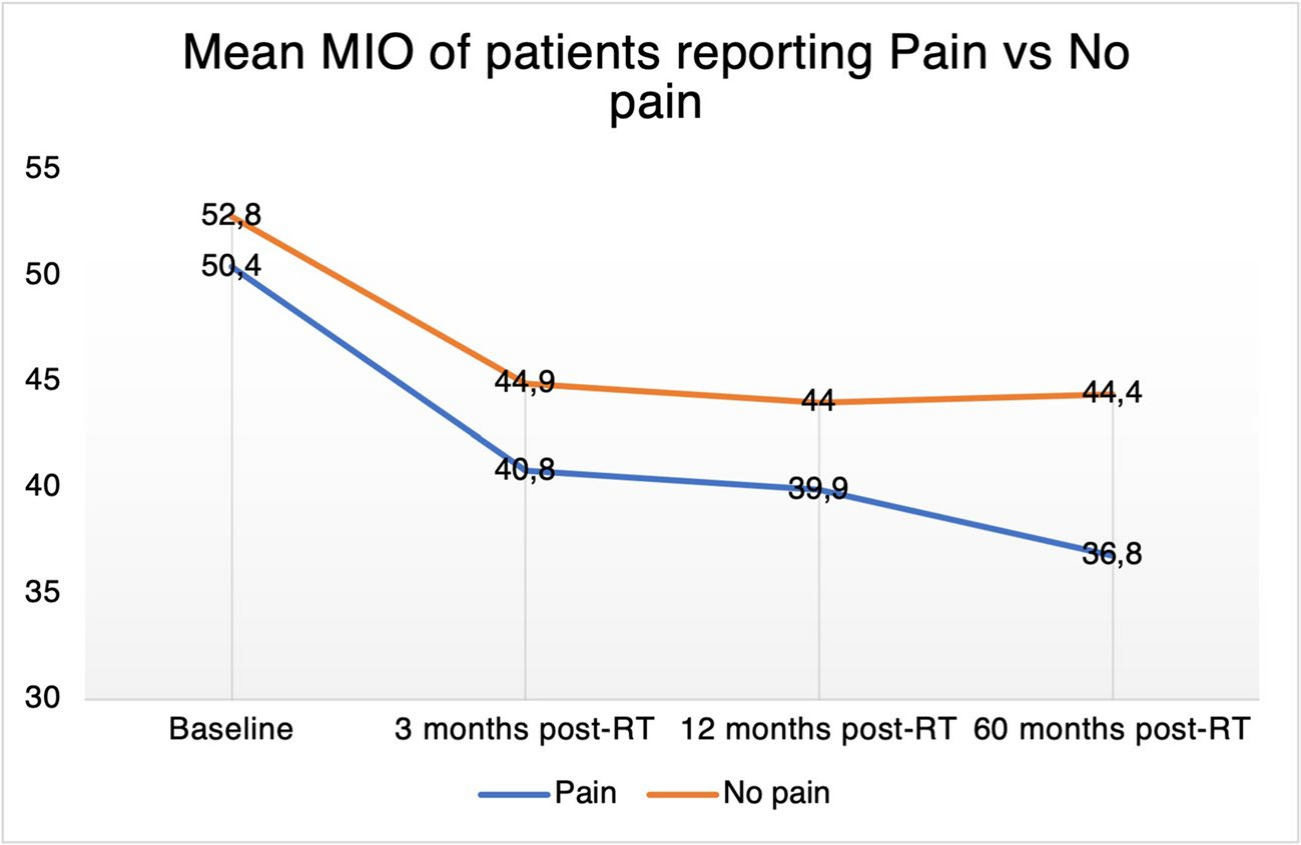
Mean MIO in millimeters for patients in the facial pain group vs no facial pain at each study time point

MIO was significantly lower in the pain group at all four time points (Fig. 1) compared to the group reporting no pain. The distribution of pain intensity on a scale of 1–7 points in the pain group is presented in Table 5. A majority of patients reported 2–3 points of 7 (i.e. mild pain) on the GTQ pain question at each time point. At baseline, 43 out of the 97 subjects (44%) in the pain group reported moderate to severe pain the last month. The corresponding figures for the 3-month, 12-month, and 60-month follow-up were 54/129 (42%), 33/90 (37%), and 15/52 (29%), respectively. The subjects reporting higher scores tended to have a lower MIO. However, the correlation between these variables was weak during the first year post-RT (*r* = 0.29) and moderate at the 5-year follow-up (*r* = 0.48).

**Table 5:** Distribution of pain intensity according to the GTQ question “Intensity of facial pain during the last month” and correlations with MIO in millimeters

		1 point †	2 points	3 points	4 points	5 points	6 points	7 points	*P* value r
Baseline		N = 97	N = 33	N = 21	N = 32	N = 8	N = 3	N = 0	
	MIO mean (95% CI)*	52.8 (52–54)	51.5 (49–54)	51.0 (48–54)	48.8 (47–51)	50.8 (42–60)	51.0 (36–66)		< 0.01 − 0.19
3 months post-RT		N = 56	N = 46	N = 29	N = 42	N = 8	N = 4		< 0.001 − 0.27
	MIO	44.9 (43–47)	42.9 (40–45)	39.4 (36–43)	40.3 (38–43)	35.0 (30–40)	42.5 (35–51)		
12 months post-RT		N = 78	N = 37	N = 20	N = 23	N = 10	N = 0		< 0.001 − 0.29
	MIO	44.0 (42–46)	39.6 (37–42)	42.4 (38–47)	38.3 (34–43)	34.5 (28–42)			
60 months post-RT		N = 74	N = 25	N = 12	N = 8	N = 6	N = 1		< 0.001 − 0.48
	MIO	44.4 (43–46)	40.0 (37–43)	35.0 (31–39)	36.4 (30–43)	29.3 (23–36)	28.0		

*The data are displayed as mean (95% CI = Confidence Interval)

^†^ 1 point = no pain, 2–3 points = mild pain, 4 points = moderate pain, 5–6 points = severe pain, 7 points = unbearable pain 29

## Discussion

This longitudinal observational cohort study describes pain and HRQL in 194 patients with HNC at multiple time points after completion of radiotherapy with curative intent and is to our knowledge the longest prospective study to date examining the association between facial pain and restricted mouth opening in this setting.

Study results show that a large proportion of treated HNC patients still suffer from facial pain long-term. Approximately 50% of the study population reported some degree of facial pain at baseline before starting oncologic treatment, which then increased to 70% at 3 months post treatment completion. This decreased to 54% at 12 months post-RT completion and finally dropped to 41% at 5 years post-RT. These results are similar to previous findings from other studies. In a cross-sectional study by Ren et al. 23, a HNC cohort of 505 survivors with a mean survivorship of 4.6 years and a history of cancer treatment of radiotherapy (81.5%), surgery (62.9%) and chemotherapy (58.1%) filled out surveys asking if they had had pain in the last week. If yes, they were asked to grade the pain on a numerical pain scale from 0 (no pain) to 10 (as severe as it could be). They were given a body map to point out where in the body they experienced the pain. 45% of HNC survivors with a median follow-up of 3 years post cancer treatment reported pain. The locations of pain were mainly reported being in the “neck and throat”, followed by “head and oral cavity” and lastly “shoulder”. In another study by Cramer et al. 21 from 2018, patients were asked to self-report grade of pain, from none to severe followed by a secondary assessment of pain as measured by the University of Washington quality of life questionnaire. The received cancer treatment of the cohort was mostly multi-modal including surgery, RT and chemotherapy alone or in combination, where 83% had received RT and 60% surgery. 45.1% of 175 HNC patients with a median of 6.6 years after diagnosis reported pain. These patients were however included from a survivorship clinic which may theoretically result in an overrepresentation of reported pain in that specific group. Furthermore, type of cancer pain is not specified in the study. A systematic review by Epstein et al. 31 reported that 50% present with orofacial pain at initiation of treatment (chemotherapy and radiation for HNC) and the number increases to 81% during therapy, dropping to 70% at the end of therapy with 36% of patients still presenting with orofacial pain at 6 months post-therapy. Pain was often assessed using validated quality of life questionnaires such as EORT QLQ-C30, EORTC QLQ-HN35, University of Washington Quality of Life questionnaire (UW-QOL), as well as visual analog scale and study specific questionnaires like Oral Mucositis Daily Questionnaire among others.

According to the results from the EORTC QLQ-C30 and HN35 in our study, the pain group also reported worse HRQL on most scales and items in both questionnaires. Hence, a clear association was seen between reports of presence of facial pain and negatively affected HRQL. The majority of other post treatment studies regarding HNC, pain and quality of life include pain in assessment of more broadly evaluated quality of life as also noted in the mentioned systematic review by Epstein et al. 29. Furthermore, prospective follow-up studies for years on the subject of pain after oncological treatment are difficult to find. This further complicates comparison of results. In the above mentioned the cross-sectional Cramer et al. study on patients with HNC following treatment, 46% of the patients reporting pain reported low overall quality of life versus 12% of those who reported to be pain-free 21. Pain was assessed in two formats, first by asking about presence and degree of pain, then pain was secondarily assessed in the UW-QOL survey.

Furthermore, patients who scored higher (worse) regarding pain, also appear to have a decreased MIO. Albeit this correlation was weak during the first year post-RT, a moderate correlation was shown towards the end of the study period. A possible explanation for this change in degree of correlation strength may be that acute treatment toxicities which are not directly linked to mouth opening, including oral mucositis, cause pain and discomfort during the first time-period post-RT 32. Factors supporting this theory may also be found in the patient-reported HRQL, where scores were worse for symptoms such as *Fatigue* (EORTC QLQ-C30), *Senses* and *Social eating* (EORTC QLQ-HN35) at 12 months post-RT compared to 5 years post-RT in the group reporting pain. A similar pattern was observed for the EORTC QLQ-HN35 single item *Dry mouth*, again potentially hinting at consequences of acute treatment toxicity.

Albeit caution should be advised when drawing conclusions regarding the causative effects of a multifactorial phenomenon such as the perception of pain, our results may point to the possibility of trismus causing pain or vice versa. In an interventional, matched control study by Andréll et al. 18, HNC patients with post-radiation trismus underwent a jaw exercise program (*n* = 50) and were compared to a matched control group who did not undergo the structured jaw exercise program (*n* = 50). Fifty-nine percent of the subjects reported facial pain before oncologic treatment and more than half (51%) of the HNC survivors with trismus reported facial pain at three years post treatment completion. The patients with HNC in the Andréll study all had post-RT trismus. The higher prevalence of pain in that study population indicates that there is an important relationship between a lower MIO among HNC survivors and the risk of reporting pain. Another important finding in the Andréll study that underlines the relationship between facial pain and trismus was that the patients undergoing the structured jaw exercise program had 9 times higher chance of being pain-free at 3 years following treatment. Nonetheless, there are numerous other potential causes of head and neck pain after oncologic treatment for HNC, including osteradionecrosis, lymphedema, burning mouth syndrome and neurological damage 33.

The strength of this study lies in its prospective design with a long follow-up period of 60 months. Furthermore, the objective measurement of MIO, the use of validated, well used patient reported outcome measures and the size of the cohort also contribute to its strength. The study may be limited by the fact that the patients who dropped out of the study had somewhat worse overall health at baseline. Recurrent HNC is an important cause of local pain and the patients in the study who developed a recurrent disease were excluded. Hence this potential cause of reported pain was minimized, possibly further potentiating the risk of survival bias. Furthermore, there might be a slight risk of recall bias considering the recall time of the primary variable in this study being 1 month. In the more recent version of GTQ, the recall time for pain has been changed to 1 week. There is also limited information regarding the use of specific pain medication or if other methods have been used for alleviating pain, as well as the exact location and cause of the facial pain.

Finally, most of the patients had received chemoradiotherapy, and no direct comparison of pain outcomes was made between those who received chemoradiotherapy and those who received radiotherapy alone. Therefore, the effect of chemotherapy versus radiotherapy alone could not be distinguished in this study.

### Clinical implications

It is clear from the study that pain is a prevalent symptom experienced by irradiated HNC patients both short-term as well as long-term. It also has a large impact on HRQL. Hence, it is of utmost importance to follow these patients over time in order to identify and alleviate pain. Trismus may very well be a contributing factor and mouth opening ability should also be measured and structured jaw exercises initiated when trismus occurs in an attempt to increase mouth opening and reduce facial pain in the long-term within the HNC population.

## Conclusions

The study findings demonstrate that facial pain was common among HNC patients before oncological treatment as well as up to 5 years post-RT. Reductions in MIO were associated with significantly more facial pain, particularly in the long term and patients who reported facial pain also reported worse HRQL compared to those reporting no pain. The degree of facial pain needs to be evaluated continuously by clinicians to ensure that other possible methods or medications for alleviations are offered to increase the HNC patients’ chance of optimal well-being.

## Data Availability

The data supporting the findings of this study are available from the corresponding author, upon reasonable request
